# The Sample Size Matters: To What Extent the Participant Reduction Affects the Outcomes of a Neuroscientific Research. A Case-Study in Neuromarketing Field

**DOI:** 10.3390/s21186088

**Published:** 2021-09-10

**Authors:** Alessia Vozzi, Vincenzo Ronca, Pietro Aricò, Gianluca Borghini, Nicolina Sciaraffa, Patrizia Cherubino, Arianna Trettel, Fabio Babiloni, Gianluca Di Flumeri

**Affiliations:** 1 Department of Anatomical, Histological, Forensic & Orthopedic Sciences, Sapienza University of Rome, Piazzale Aldo Moro 5, 00185 Rome, Italy; vincenzo.ronca@uniroma1.it; 2 BrainSigns srl, Via Lungotevere Michelangelo, 9, 00192 Rome, Italy; pietro.arico@uniroma1.it (P.A.); gianluca.borghini@uniroma1.it (G.B.); nicolina.sciaraffa@uniroma1.it (N.S.); patrizia.cherubino@uniroma1.it (P.C.); arianna.trettel@brainsigns.com (A.T.); fabio.babiloni@uniroma1.it (F.B.); gianluca.diflumeri@uniroma1.it (G.D.F.); 3 Department of Molecular Medicine, Sapienza University of Rome, Viale Regina Elena, 291, 00161 Rome, Italy; 4 Department of Computer Science, Hangzhou Dianzi University, Hangzhou 310018, China

**Keywords:** applied neurosciences, EEG, HR, GSR, sample size, signal processing

## Abstract

The sample size is a crucial concern in scientific research and even more in behavioural neurosciences, where besides the best practice it is not always possible to reach large experimental samples. In this study we investigated how the outcomes of research change in response to sample size reduction. Three indices computed during a task involving the observations of four videos were considered in the analysis, two related to the brain electroencephalographic (EEG) activity and one to autonomic physiological measures, i.e., heart rate and skin conductance. The modifications of these indices were investigated considering five subgroups of sample size (32, 28, 24, 20, 16), each subgroup consisting of 630 different combinations made by bootstrapping n (n = sample size) out of 36 subjects, with respect to the total population (i.e., 36 subjects). The correlation analysis, the mean squared error (MSE), and the standard deviation (STD) of the indexes were studied at the participant reduction and three factors of influence were considered in the analysis: the type of index, the task, and its duration (time length). The findings showed a significant decrease of the correlation associated to the participant reduction as well as a significant increase of MSE and STD (*p* < 0.05). A threshold of subjects for which the outcomes remained significant and comparable was pointed out. The effects were to some extents sensitive to all the investigated variables, but the main effect was due to the task length. Therefore, the minimum threshold of subjects for which the outcomes were comparable increased at the reduction of the spot duration.

## 1. Introduction

In the field of behavioural neurosciences, as well as in every scientific discipline, sample size definition is a crucial factor for ensuring the reliability and accuracy of the results obtained in a study, as well as the replicability of the study itself [1]. In fact, the statistical power and the reproducibility of a study are strictly related to the number of participants included in the analysis [2]. In order to prevent and mitigate consequences of a poor statistical power, it is generally recommended to estimate the proper sample size. To this end, different studies recently tried to propose methods to easily calculate the required sample size while designing the experimental protocol [3,4,5,6,7,8]. Additionally, sometimes it is not only strictly a problem of sample size, intended as the number of participants, but also the concerns of trial numbers, i.e., the number of tasks and repetitions should be carefully considered [9]. Backe and colleagues pointed out the relation between the number of trials necessary to perform studies involving human participants and the sample size. They proposed a tool based on power contours for calculating the optimal combination of trials and number of participants [10]. Additionally, Guttmann-Flury [11] pointed out a method for a priori sample size determination based on Montecarlo simulations on Electroencephalographic (EEG) data. Therefore, the inclusion of an adequate number of participants in neuroscientific studies is an important issue, and some authors reported alarming data about the potential risks of employing a small sample size [1]. This is the case of Button and colleagues [2], who claimed that many of the works published in neuroscientific domain are not reproducible due to their small sample size and the low statistical power associated with false-positive effects counted in the results. The reduction of the sample size is denounced as a deliberate action, driven by the aim of researcher or organisations which want to increase the prestige associated with the publishing of their works at any cost [12]. A smaller sample size, in these cases, is chosen to undermine the statistical power of the study, enhancing the presence of false-positive effects which endorse the desired results. 

Though this behaviour must be countered, there are cases in which the reduction of the sample size is necessary for organisational and logistic needs. In fact, thanks to the increasing shared knowledge and recent scientific evidence produced by the neuroscientific community, behavioural neuroscience is experiencing an exponential growth in terms of applied research [13,14,15]. Recently, new terms have been coined, such as Neuroergonomics, Neuromarketing, Neuroaesthetics, and others, to include the new neuroscientific disciplines that aim at investigating neurophysiological correlates of humans’ behaviour at work [16,17,18], including driving a car [19,20,21,22,23,24], watching television [25,26,27], having a sensorial experience [28,29,30], and in any other activity of daily life. In the context of applied neurosciences, when physiological signals are acquired in real environments, several factors work against the ease of participant recruitment. The whole experimental session of each subject requires time, and the operators have to welcome the participant, explain the protocol, ask for informed consent, place the devices in the correct way, ensure the quality of the signals, and finally start the experimental tasks during which biosignals are recorded. Therefore, participants should account for taking one or two hours off from their working day for taking part in the experiment. At the same time, expert operators should be available to spend several days to perform the experiment, usually outside their working place (i.e., outside the laboratories). Furthermore, if the study is conducted in real environments, out from the labs, it is necessary to concentrate all the recordings in few days because generally the facilities that host the experiments are not available for long. Therefore, the number of slots at disposal to screen each participant is not so high. In the last period, moreover, the on-going Coronavirus Disease 2019 (COVID-19) pandemic [31] made participant recruitment even more difficult. During the study, both participants and operators take the risk of reaching the place in which the experiments are performed, they must carefully observe all the sanitising practices, avoid contacts, and maintain social distancing. The time spent on respecting these procedures renders the duration of the protocol even longer than usual. The reduction of the sample size, in these cases, would allow for the performing of experiments without compromising the experimental protocols and ensuring at the same time the good sanitizing practices and any other health preventive measure.

In this context, premising that the best practise is to include an optimal sample size, it is interesting to explore what error affects the outcomes of a study when researchers rely on a lower number of subjects. In other words, in those cases when for different reasons it is not possible to enlarge the sample size anymore, the researchers should be at least aware of the potential error affecting the results in order to interpret them properly. With this aim, the present study took into consideration a case study coming from the neuromarketing domain. It was analysed through an existing dataset of thirty-six participants while performing a neuromarketing experimental protocol in order to explore the modification of three neurophysiological indexes, frequently employed in applied neurosciences, when the participants decrease, pointing out the conditions for which the indices remain comparable, and quantifying the tendency of measurements variability and related error depending on the sample size. Five subgroups of subjects’ numerousness (i.e., sample size) from the total sample of participants (36, hereafter ‘Reference population’) were considered: 32, 28, 24, 20, and 16 subjects respectively (sub-groups). In particular, each subgroup consisted of 630 different combinations (since 630 is the maximum number of different combinations of 32 elements from a set of 36) of the whole ‘Reference population’. The outcomes associated with the entire population were considered as reference since 36 is within the range of sample sizes generally involved in this kind of study. This number was deemed appropriate to assume the results were reliable, i.e., representative of the theorical population, in line with the scientific literature [32]. These subgroups were chosen since: (i) in the literature there is a large consensus considering [16,17,18,19,20,21,22,23,24,25,26,27,28,29,30,31,32] the optimal range of participants in neuroimaging studies [33]; (ii) in order to perform Analysis of Variance, the number of cases (i.e., 630) has to be much higher than the number of groups (i.e., five). The experimental task consisted of watching four TV spots with different lengths (30, 30, 20, and 15 s) during which electroencephalographic (EEG) signal and autonomic measures, i.e., heart rate (HR) and galvanic skin response (GSR), were acquired. The data analysis explored the correlation and the error between the outcomes for the total sample size and the considered subgroups of subjects. Furthermore, the data distribution over the experimental sample was explored in terms of standard deviation of the indexes, for each second, at the participants’ reduction. The hypothesis at the base of this study was to find a decrease of correlation as well as an increase of error and data dispersion moving from 32 to 16 subjects with respect to the total sample size (36 subjects). In addition, the data analysis was conducted considering three influencing factors: the kind of index, the task, and the time (i.e., task length), assuming that they could affect the outcoming modifications at the sample size reduction.

In conclusion, all the possible sub-sample sizes from 34 up to 10 were considered in order to model the decreasing curve of sample size-correlation relationship. 

Before going ahead with the methods, it is important to clarify that the analysis was focused on investigating the alterations induced by sample size reduction on neurophysiological indicators averaged on the whole available sample (group analysis), without taking into consideration the sensitivity of the resulting indicators (i.e., the indicators computed on smaller samples) with respect to the phenomenon itself (i.e., if the sample size was reduced the probability of wrongly rejecting the null hypothesis—type I error—or wrongly accepting the null hypothesis—type II error—would increase or decrease). This concern is addressed in Section 4.

## 2. Materials and Methods

### 2.1. Experimental Sample and Design

The dataset analysed in this study included EEG, GSR, and PPG (photopletismographic) signals acquired from 36 subjects (37.39 ± 10.67 years old), gender balanced, recruited on a voluntary basis. These subjects signed the informed consent and the related information sheet, in which the study was explained, before participating in the experiment. The experiment was conducted following the principles outlined in the Declaration of Helsinki of 1975, as revised in 2000, and it received the approval of Sapienza University of Rome ethical committee (nr. 2507/2020). 

The task consisted of watching a TV video, almost 12 min in length, based on a documentary and with two commercial breaks in the middle. Such breaks consisted of twelve different TV commercials selected as experimental stimuli, six per break, presented in a randomised order. This means that the same spot was seen by the participants in different moments of the experience in order to avoid any habituation effect. Subjects were comfortably seated in front of the screen during the entire protocol. The documentary was chosen to be relaxing and emotionally neutral [34], in contrast with the high emotional and engaging contents of TV advertising, and it was used as individual baseline. The task was designed for neuromarketing research, and thus one of the new branches of applied neuroscience [26,35] as depicted in the introduction, with the aim of evaluating the response to some target advertising. However, for the purpose of the present study, only four commercials over twelve were considered in the analysis. Assuming that larger spot lengths would favour correlation analysis, we took the longer (*t* = 30 s) spots. Actually, 30 s is the most common time duration employed for TV commercial advertising (source: Nielsen Report, 2013 [36]). Then, we selected two shorter spots (20 and 15 s) taking care to select non-contiguous ones in order to have four spots distributed along the whole commercial break.

### 2.2. Neurophysiological Data Recording 

The cerebral activity was recorded by means of a portable EEG system (BEmicro and Galileo software, EBneuro, Firenze, Italy). Ten Ag/AgCl electrodes were placed according to the 10–10 International System, at the following channels: Fp2, Fpz, Fp1, AF7, AF5, AF3, AFz, AF4, AF6, and AF8, all referred to the earlobes (REF channel). EEG activity was collected at a sampling rate of 256 Hz while the impedances were kept below 10 kΩ by employing conductive gel to adapt them. The raw signals were fully analysed offline through MATLAB software (MathWorks Inc., Natick, MA, USA). The power-supply interference was removed with a notch filter at 50Hz. Moreover, signals were bandpass filtered by a 5th-order Butterworth filter [2,30] Hz) in order to remove interferences in frequencies not of interest. Independent component analysis (ICA) was then performed, in particular by employing the SOBI algorithm [37], to remove Independent Components related to ocular and muscular activity. On average, 2 ± 1 components were manually selected and removed by the same neurophysiology expert. The reconstructed EEG signal was then segmented into 1-s-long epochs with 0.5 s of overlap in order to avoid any “boundary effect”, and three additional criteria for detecting artifacts based on the signals’ amplitude and trend [38,39] were applied in order to remove those portions of data still affected by artifacts that had not been corrected before. 

At the beginning of the EEG recording, the participants were invited to close their eyes for 60 s. Such a time interval was then employed for the calculation of the individual alpha frequency (IAF), i.e., the peak of the signal power spectrum within the traditional alpha frequency range (7–12/13 Hz). According to Klimesch and Doppelmayr [40], the IAF was then used to define the individual EEG frequency bands of each participant as a function of the IAF itself ([IAF–x, IAF + x] Hz). In particular, for the purposes of this study two EEG frequency bands have been investigated, the whole alpha [IAF–4Hz, IAF + 2 Hz] and the upper alpha [IAF–2Hz, IAF + 2 Hz] [41]. Then, the EEG signals for each subject were filtered in these specific sub-bands and the global field power (GFP) [42] was computed in order to obtain a synthetic indicator of the synchronous de-/activation of brain cortical areas. 

In terms of autonomic measures, Galvanic Skin Response (GSR) and Photopletismographic (PPG) signal were recorded with a Shimmer3+ (Shimmer Sensing, Ireland) device applied to the non-dominant hand. Two electrodes were placed on the palmar side of the middle phalanges of the second and third fingers according to scientific best practice [43], with the PPG sensor over the thumb. These signals were acquired with a sampling rate of 64 Hz. From the PPG signal, the Heart Rate (HR) was obtained by means of the Pan-Tompkins algorithm [44]. On the other side, the tonic component of the skin conductance (Skin Conductance Level, SCL) was estimated with the LEDAlab software and the Continuous Decomposition Analysis [45]. 

At this point, from the available biosignals pre-processed as described so far, three indices with different natures were considered in the analysis.

1. Index 1: general descriptor of the frontal cerebral activity. It was computed as the inverse of the upper-alpha GFP over all the frontal electrodes. It is generally related to the attentional processes [46,47];
2. Index 2: descriptor of the frontal alpha asymmetry [48]. It was computed as the difference between the GFP over right (Fp2, AF4, AF8) and left (Fp1, AF3, AF7) electrodes. Such a “neurometric” is frequently employed in EEG studies [49,50], and it is known as the Approach-Withdrawal index [51];
3. Index 3: descriptor of the autonomic response, namely, the emotional index (EI), computed as the combination between the SCL and the HR measures, as described by Vecchiato and colleagues [52].

Each Index is a time-series with a time resolution of 1 s for each task and for each subject. In general, individual indexes were then averaged among the subjects in order to estimate the population index. 

### 2.3. Data Analysis and Statistics

Among all the 12 tasks (i.e., TV spots), four in particular were chosen for the analysis: two 30-s-long (the maximum length of the tasks in the present protocol) in order to verify the task-independency of the results, one 20-s, and another 15-s-long in order to also investigate the effect of task duration. At first, for each of the selected spots and for each of the three indices, 25 subgroups of subjects’ numerousness (from 10 to 34 participants) taken from the 36 participants at the study were considered in the analysis. For each of these subgroups, 630 different random combinations of subjects were selected using a bootstrap, from the initial population. It was carried out to avoid any possible effect associated to the choice of subjects. The number of combinations for each subgroup (630) was chosen since it is the maximum number of possible combinations considering a subset of 34 subjects from the initial sample of 36 (Equation (1)).

(1) $$C_{\left(36,34\right)}=\;\frac{36!}{\left(34!\left(36-34\right)!\right)}=630$$

For coherence, this number of combinations was extended to the other 24 subgroups of subjects by randomly selecting 630 out of all the possible combinations for that specific case.

Keeping in mind that for each task, each index corresponds to a t-long (with *t* = 30, 20, 15) vector (i.e., the population index ‘*v*’), for every subgroup, for each spot, and for each index, we computed:

1. Group-mean values of the index (function of *t*, i.e., the task duration), for each of the 630 combinations. We so obtained 630 vectors ‘*v630*’ (thus resulting in a matrix 630 x *t*);
2. Pearson correlation between each ‘*v630*’ (630 x *t*) and the vector ‘v’ (1 x *t*), containing the mean values of the index computed over the entire population (36 subjects);
3. Mean Squared Error (MSE) to describe the error committed considering each ‘*v630*’ rather than ‘*v*’ along each task (within-task variability):
  (2) $$MSE=\frac{\sum_{i=1}^{t}{\left(v630_{i}-v_{i}\right)}^{2}}{t}$$

In this formula ‘*v630*’ represents the vector of the observed values while ‘*v*’ is assumed as the vector of predicted values (i.e., the unique possible vector of each index if considering the full population).

4. The standard deviation of the 630 values assumed by the vectors ‘*v*630’, for every second of the task itself (between-groups variability):
  (3) $$STD=\sqrt{\frac{\sum_{n=1}^{630}{\left(v630_{n}-\bar{v630}\right)}^{2}}{630}}$$

The effect of the sample size reduction was explored considering the variables described in the previous section.

In order to ensure the statistical power of the analysis, only five subgroups with a steady reduction of the sample size were considered. Specifically, they were subgroups of 32, 28, 24, 20, and 16 subjects.

**Figure 1:**
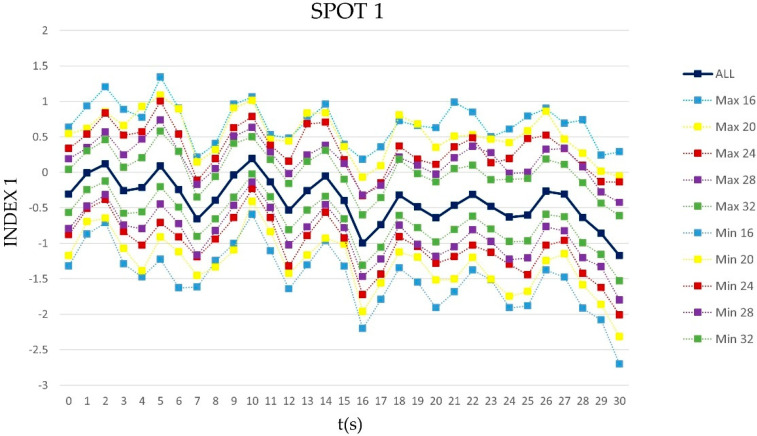
The graph represents the trend of ‘*v*’, the mean value of Index1 computed over the entire population (36 subjects) during SPOT1 (ALL). The dashed lines show the trend of the maximum and minimum values (Max, Min) of the index among the ‘*v630*’ for each second, in each subgroup of subjects (32, 28, 24, 20, 16).

In the figure below (Figure 1), it is possible to visually appreciate, for a representative spot and index, the increasing variability of the index itself when decreasing the sample size. In fact, they represented the maximum (Max) and the minimum (Min) values of the index among the 630 combinations of the n-th sample size (n = 32, 28, 24, 20, 16), i.e., the ‘*v630*’ vectors with respect to the ‘*v*’ one (full sample size, i.e., ALL).

The statistical analysis was performed on these data to assess the differences among the five sub-groups. At first, the Pearson correlation between each ‘*v630*’ with respect to ‘*v*’ was conducted for each subgroup of subjects and the number of significant correlations were reported. The *p*-value for these correlations was corrected through the Bonferroni method in order to avoid any improper deduction due to the type I error, the likelihood of which is higher with greater numbers of repeated statistical tests carried out. In particular, being that the multiple comparisons were equal to 630, the test was deemed significant for *p* < 8 × 10^−5^ [53]. The Shapiro Wilk test [54] of normality demonstrated that data (the Rho correlation coefficients, MSE, and STD values) were not Gaussian; hence, the non-parametric Friedman test [55] was performed to assess the difference between groups. Specifically, the subgroups of subjects (32, 28, 24, 20, 16) were considered as within factors for the analysis run over the Rho values resulting from the correlation analysis, the MSE and the STD values. The analysis was performed for each of the three considered indices and each of the four selected spots. Nemenyi post-hoc test [56], specifically conceived for non-parametric repeated measures ANOVA (i.e., the Friedman test), was applied to further analyse the significant effects and interactions.

In conclusion, the Rho correlation coefficient was computed and summarised for each index for each task for all the possible sub-sample sizes (from 34 up to 10) in order to visually highlight the decreasing curve of sample size-correlation relationship.

## 3. Results

### 3.1. The Effect of the Index

The analysis of the first spot (SPOT1) was conducted exploring the effect of the sample size reduction on the three different indices.

**Figure 2:**
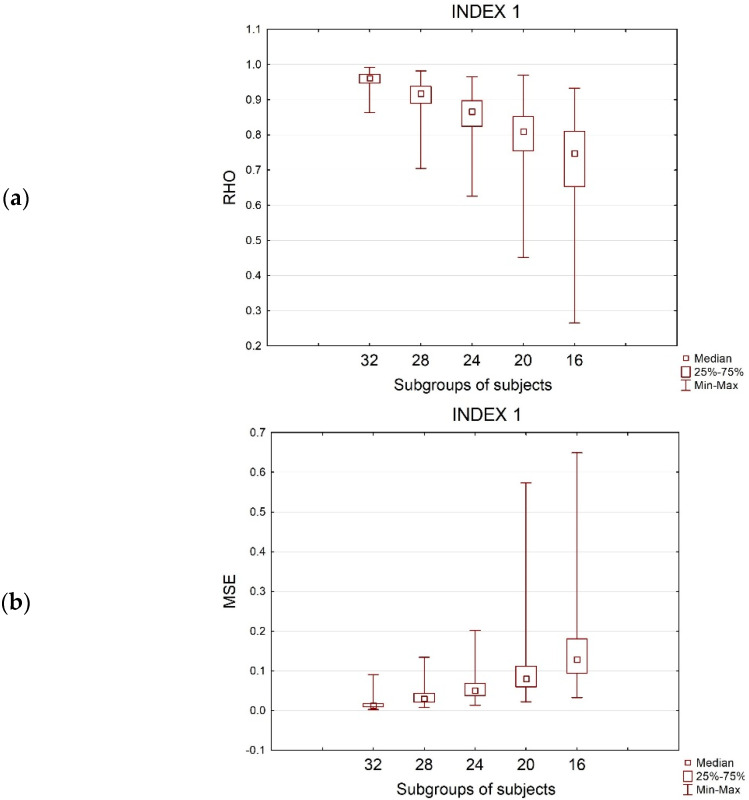

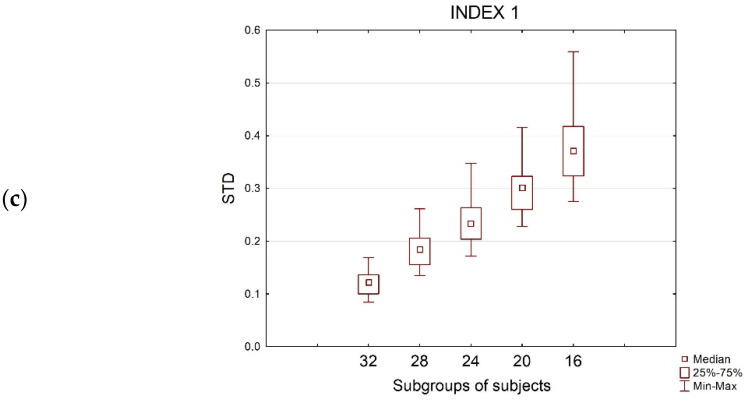
The box plots represent the effect of the subgroup of subjects on (**a**) Rho values, (**b**) MSE values, and (**c**) STD values, computed for Index 1, during the SPOT 1.

Concerning Index1, EEG frontal desynchronization, the correlation analysis showed that ‘*v630’* significantly correlated with the vector ‘*v’*, associated with the entire population, considering the subgroups of 32 and 28 subjects. Reducing the sample size to 24, to 20, and to 16, two correlations over 630, 33 over 630, and 151 over 630 resulted as respectively not significant (*p* > 8 × 10^−5^). The Friedman test evidenced a significant decreasing effect of the mean rho values depending on the sub-groups, Figure 2a (Friedman chi-squared = 1868, *p*-value < 2.2 × 10^−16^). The post-hoc analysis showed that rho significantly decreased from 32 to 16 subjects, assuming different values for each subgroup (*p* < 0.05).

Additionally, the MSE showed dependency from the subgroups of subjects, Figure 2b (Friedman chi-squared = 1932.3, *p*-value < 2.2 × 10^−16^): it was significantly lower for 32 subjects, increasing with the sample size reduction. The highest error was committed considering 16 subjects instead of 36. All the groups were significantly different as showed by the Nemenyi test.

The analysis of the STD evidenced the effect of the groups, Figure 2c (Friedman chi-squared = 124, *p*-value < 2.2 × 10^−16^). It was lower for 32 subjects, increasing with the reduction of the sample size. The post-hoc analysis demonstrated that STD was significantly different for each subgroup.

Concerning Index2, EEG frontal asymmetry, all the correlations were significant for the subgroups 32 and 28 (*p* < 8 × 10^−5^). Concerning the subgroups of 24, 20, and 16 subjects, 13 over 630, 92 over 630, and 237 over 630 correlations were respectively not significant. The Friedman analysis on rho, MSE, and STD showed the effect of the groups, with a significant increase of rho (Friedman chi-squared = 1868, *p*-value < 2.2 × 10^−16^), a significant increase of MSE (Friedman chi-squared = 1932.3, *p*-value < 2.2 × 10^−16^), and a significant increase of STD from 32 to 16 subjects (Friedman chi-squared = 124, *p*-value < 2.2 × 10^−16^). The post-hoc Nemenyi test evidenced that all the subgroups assumed different values of rho, MSE, and STD.

Considering Index3, Autonomic response, for every subgroup, only the subgroup 16 presented 12 not-significant correlations over 630 (*p* > 8 × 10^−5^). The Friedman analysis on rho, MSE, and STD showed the effect of the groups, with a significant increase of rho (Friedman chi-squared = 2235.5, *p*-value < 2.2 × 10^−16^), a significant increase of MSE (Friedman chi-squared = 2232.1, *p*-value < 2.2 × 10^−16^), and a significant increase of STD from 32 to 16 subjects (Friedman chi-squared = 124, *p*-value < 2.2 × 10^−16^). The post-hoc Nemenyi test evidenced that all the subgroups assumed different values of rho, MSE, and STD.

The analysed variables for SPOT1 and the three indexes are summarised in Table 1.

### 3.2. The Effect of the Task

The analysis on SPOT2 (Table 2), with the same length of SPOT1 but different content, showed that all the correlations between the ‘*v630’* and ‘*v’* were significant for the subgroups 32, 28, and 24, considering Index1 (*p* < 8 × 10^−5^), while for the subgroups 32 and 28 for Index2 and Index3. The analysis evidenced that reducing the sample size at 24:10 and 49 over 630 correlations were not significant for Index2 and Index3, respectively. For a sample size of 20 subjects, 8, 90, and 138 over 630 correlations were not significant for the three indices, respectively. Moving on, 16 subjects, 49, 215, and 290 over 630 were not significant for the three indices, respectively. Friedman test on rho, MSE, and STD revealed a significant effect of the groups for the three indices, showing rho decreasing (Index1: Friedman chi-squared = 1879.4, *p*-value < 2.2 × 10^−16^; Index2: Friedman chi-squared = 1800.9, *p*-value < 2.2 × 10 ^−16^; Index3: Friedman chi-squared = 1888.1, *p*-value < 2.2 × 10^−16^), MSE increasing (Index1: Friedman chi-squared = 2124.8, *p*-value < 2.2 × 10^−16^; Index2: Friedman chi-squared = 2151.2, *p*-value < 2.2 × 10^−16^; Index3: Friedman chi-squared = 2182.7, *p*-value < 2.2 × 10^−16^), and STD increasing (Index1: Friedman chi-squared = 124, *p*-value < 2.2 × 10^−16^; Index2: Friedman chi-squared = Friedman chi-squared = 124, *p*-value < 2.2 × 10^−16^; Index3: Friedman chi-squared = 124, *p*-value < 2.2 × 10^−16^). The post-hoc test showed that all the comparisons between groups were significant.

### 3.3. The Effect of the Time

To explore the influence of the task time duration, or rather the length of the spot in this case, the last analysis was conducted on the number of significant correlations found for SPOT3 and SPOT4, respectively 20 and 15 s long (Table 3). The MSE and STD were not considered in this part of the study because they do not depend on the time. The analysis of SPOT3 showed that all the correlations were significant for the subgroups of 32 and 28 subjects for both Index1 and Index3 (*p* < 8 × 10^−5^). Concerning Index1, 12, 53, and 136 correlations over 630 were not significant in subgroups 24, 20, and 16, respectively, while for Index3, 2, 63, and 216 correlations were not significant in those subgroups. Considering Index2, the number of not-significant correlations increased from the subgroup of 28 subjects in which 23 correlations were not significant, while 115, 290, and 408 over 630 correlations were not significant for the subgroups of 24, 20, and 16 subjects, respectively. Friedman test on rho values revealed a significant effect of the groups for the three indices, showing a rho decreasing related to the reduction of the sample size (Index1: Friedman chi-squared = 1712.1, *p*-value < 2.2 × 10^−16^; Index2: Friedman chi-squared = 1586.3, *p*-value < 2.2 × 10^−16^; Index3: Friedman chi-squared = 2017.2, *p*-value < 2.2 × 10^−16^). Post-hoc test showed that all the comparisons between groups were significant. The analysis of SPOT4, showed that all the correlations were significant for the subgroups of 32 subjects, for both Index1 and Index3 (*p* < 8 × 10^−5^). Concerning Index1 63, 214, 335 and 464 correlations over 630 were not significant in subgroups 28, 24, 20 and 16 respectively, while for Index3, 19, 122, 296 and 423 correlations were not significant in those subgroups. Considering Index2, the number of not-significant correlations increased, already from the subgroup of 32 subjects in which 7 correlations were not significant, while 82, 203, 342, 450 over 630 correlations were not significant for the subgroups of 28, 24, 20 and 16 subjects, respectively. Friedman test on rho values revealed a significant effect of the groups for the three indices, showing rho decrease related to the reduction of the sample size (Index1: Friedman chi-squared = 1510.9, *p*-value < 2.2 × 10^−16^; Index2: Friedman chi-squared = 1263, *p*-value < 2.2 × 10^−16^; Index3: Friedman chi-squared = 1617.6, *p*-value < 2.2 × 10^−16^). The post-hoc test showed that all the comparisons between groups were significant.

The number of significant correlations for the three indices and the four considered spots are summarised in Table 4.

### 3.4. Rho-Sample Size Relationship

**Figure 3:**
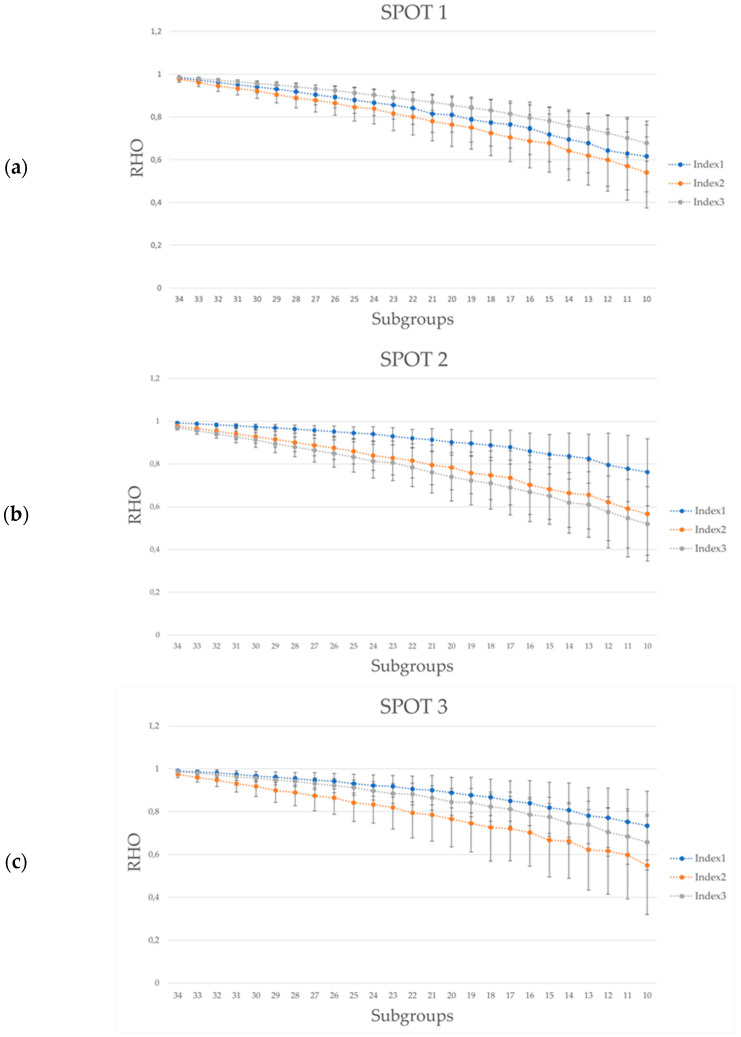

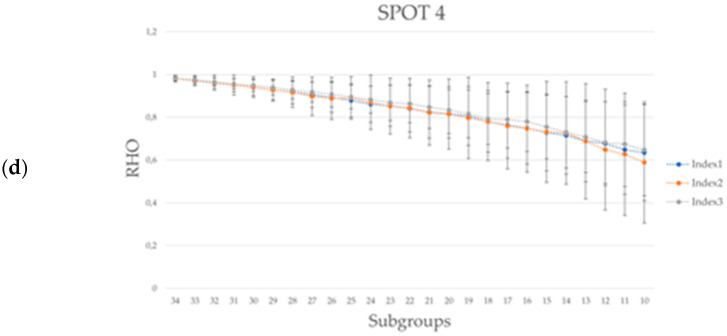
Graphical representation of the trend of rho correlation coefficient at reducing the sample size (subgroups) with respect to the ‘full population index’ (36 participants). Tasks (**a**) and (**b**) were 30 s long, Task (**c**) 20 s, and Task (**d**) 15 s long.

In conclusion, the average value of the Rho correlation coefficient between ‘*v630*’ and ‘*v*’ vectors, and its standard deviation among all the 630 combinations, were computed for all the possible sample sizes, from 34 up to 10 (generally considered as the minimum acceptable sample size) in order to model the function of the relationship between the correlation with the whole “Reference population (36 subjects) index” and the indexes resulting from reducing the sample size. Represented below (Figure 3) is the graphical representation of such a function for each spot for each index.

## 4. Discussion

In scientific literature, the reduction of the sample size is a much-debated topic. Despite many authors claiming the importance of including an optimal number of participants, applied research and even more studies performed in real contexts could be limited by several constraints, and so it could become problematic to involve large experimental samples of participants. After all, in the field of EEG research, studies published in peer-reviewed journals do not have the same sample size, even when they consider the same topic. For instance, Xu and Zhong, in their review on the EEG in educational research [57], pointed out that over the 22 screened papers, the sample size varied from 80 to 5 subjects, undermining the study outcome’s interpretability and replicability. The present research aimed to investigate the modifications of the outcomes of a study in the context of applied neurosciences when the sample size was reduced. An initial sample of 36 subjects was considered as reference. This number was deemed appropriate to represent the sample since it is around the size of participants generally involved in this kind of study. This is confirmed by the review of Bazzani and colleagues [32], who reported that, over studies that used advertisings as stimuli, 33 was the average number of recruited participants. Five subgroups of subjects were randomly chosen 630 times (each n-th combination was different from the others) and the related outcomes were compared to the condition in which all the subjects were included. The first factor considered in the analysis was the dependency from the index, or rather whether the sample size reduction had different effects on indices with different natures (general EEG activity, ‘neurometric’, intended as a combination of EEG activity features, and autonomic biosignals-related metric). Considering the targeted task, thirty seconds long, and comparing the effects found on rho, MSE, and STD in response to the decreasing of the included sample, we observed that the patterns associated to the three indices were comparable. As expected, rho significantly decreased while the MSE and STD significantly increased, moving from 32 to 16 subjects for all the indices. This latter result is particularly relevant since in scientific literature, while dealing with research outcomes’ discussion and their statistical power, effect size is generally represented through the Cohen’s d coefficient [58], computed as the ratio between the ‘µ’ mean value of the effect, and the ‘σ’ standard deviation of the measures over the sample. Cohen [58] divided effect sizes into large, medium, and small, where large effect sizes are about one (the mean has the same size as the standard deviation), medium effect sizes are about a half, and small effect sizes are a quarter, while under one eighth, the effect sizes can be considered trivial [33]. This implies that with lower sample sizes, even if the effect “amplitude” remains comparable (i.e., the ‘µ’), standard deviation (i.e., ‘σ’) increasing would negatively affect the effect size.

The number of significant correlations showed that, from 32 to 24 subjects, most of the comparisons were significant for all the three indices (Table 4). Therefore, from the first analysis, the reduction of the sample size from 36 to a maximum of 24 subjects seemed to generate comparable outcomes, independently from the nature of the considered index, committing an acceptable error (<0.1 on indexes with the 95% of distribution ranging between –3 and 3). During the second part of the study, we explored whether the findings remained consistent when the analysis was performed on a different task with same length (i.e., SPOT2). In this phase we pointed out that during SPOT2, rho, MSE, and STD values followed the same patterns observed in SPOT1: rho significantly decreased while MSE and STD significantly increased when the participants were reduced, for all the three indices. Furthermore, most of the correlations were significant considering the same threshold of 24 subjects, and the maximum deviation from 630 was observed in Index3, for which the decrease of significant correlations was equal to 5.1%. Hence, we concluded that the observed results were independent from the task when considering a spot of 30 s. The last factor investigated in the study was the effect of the time. In this phase, only the correlation was explored in the analysis since MSE and STD are not time-dependent variables. Considering spots shorter than 30 s, the correlation still decreased with the reduction of the participants but, contrarily to the SPOT1 and SPOT2, the threshold for which all the correlations were significant changed. Considering SPOT3, 20 s long, the threshold of significant correlation was 28 subjects for Index1 and Index3 but not for Index2, for which in 3.6 percent of cases (23/630), the correlation resulted as not significant. For SPOT4, the threshold increased for all the three indices: the analysis of the significant correlations showed most of the combinations were significantly correlated until 32 subjects instead of 24. These findings suggest that the modification of the outcomes related to the reduction of the sample size depended on the time, or rather on the length of the task employed in the study. It has been highlighted that the number of subjects for which the outcomes remained comparable increased with the decreasing of the spot duration, i.e., in general of the task.

If looking at the distribution of the Rho coefficients with respect to the different sample sizes (Figure 3), it has to be taken into consideration that in scientific literature the range 0.5–0.7 is considered as ‘Moderate correlation’, the range 0.7–0.9 as ‘High correlation’, and the range 0.9–1 as ‘Strong correlation’ [59]. Therefore, we can notice, independently from the index and the task length under 30 subjects, the correlation values moved from strong to high correlations, while under 18–20 subjects from high to moderate correlations, i.e., the probability that a subset of, for instance, 16 participants will represent the population behaviour over time is lower. This result is relevant considering that the statistical errors matter. As it is well known, statistical errors are classified in ‘Type I error’, i.e., the mistaken rejection of the null hypothesis (“false positive”), and ‘Type II error’, i.e., the mistaken acceptance of the null hypothesis (“false negative”). As stated in the introduction, this topic was not the focus of the study, since the experimental question was if the trends (significant or not) revealed by the study results coming from smaller samples are actually correlated with the “Reference population” behaviour (where ‘Reference population’ stands for the ideal case of the 36-sample dataset assumed as representative of the theorical population). Of course, the reduction of the sample size will undoubtedly increase the probability of a type II error, as commonly agreed upon in the literature and supported by our results (data standard deviation increasing with sample size decreasing). However, our results also shed light on the moderate correlation between a large sample and small sub-samples, meaning that it is not implicit that small samples are always representative of the population behaviour, thus increasing the probability of committing even type I error. In any case, the latter sentence has to be intended as a mere deduction, and was not demonstrated by the present study.

Therefore, it can be concluded that, as assumed, the correlations between the outcomes associated with the entire ‘Reference population’, considered as 36 observed samples, and the outcomes computed for different subgroups of subjects, decreased with the reduction of the sample size. As expected, the errors committed with the decreasing of the sample size increased as well as the dispersion of the data. However, it was possible to determine a threshold of subjects, above which almost all the comparisons were significant, the outcomes remained comparable, and the committed errors and the data dispersion were acceptable. This threshold was 24 subjects when the task lasted 30 s and 28 when the task length was reduced, even 32 when reduced up to 15 s. Further investigations will be necessary to explore whether these findings are replicable in the same and in other contexts, considering different kind of datasets. In addition, also the effect of the time needs to be examined further; for instance, it could be interesting to analyse whether the outcomes change considering different parts of the same task, or maybe with respect to specific events as well as in conditions discriminability, a common problem when employing machine-learning techniques [60,61]. In fact, in this study, time-varying functions (with a time resolution of 1 s) were employed, but in some research there is no need of achieving such a high time resolution of assessment: in some experimental protocols, lower time resolutions are enough (from some seconds to even minutes) [18,62,63]; alternatively, in other protocols, EEG indexes are employed as large averages on the entire task in order to investigate task discriminability [64,65]. In these cases, the lower time resolution would decrease the intra-subject variability, thus impacting the group average of the indexes, and therefore sample size variation could have different effects. Another common neuroscientific experimental approach envisages the analysis of Event-Related Potentials (ERPs) [24,66]; in this case, temporal resolution is even higher (milliseconds), but signal-to-noise ratio is increased by increasing the number of repetitions and computing multiple averages over them. Again, also in this kind of application, sample size variation could have different effects; therefore, neuroscientific field encloses several methodologically different applications.

This is the main limitation of the present research: the results of this study have to be considered as strictly related to this case study. The consistency of the results among the different tasks in the experiment and the outcomes of the research need to be investigated with larger samples (more than 36) and in other contexts to be generalisable. This should encourage further research to generalise the outcomes, i.e., ideally (but probably it would sound utopistic) to define a mathematical model able to predict the proper sample size of an experiment as a function of for example experimental design, time resolution, type of indicators, and so on. In addition, another topic that should be investigated more in depth is that one related to the probability of committing statistical errors. As discussed before, this study demonstrated why the probability of committing type II errors increases when decreasing the sample size. However, this study also hinted at the possibility of inferring wrong conclusions and committing type I errors with small sample sizes.

In conclusion, this study, of course, has not to be interpreted as an encouragement to reduce sample size in scientific research. However, in a context when participants recruiting is limited for several reasons, the present study aimed to provide an overview of sample reductions consequences in order to shed light on this concern when discussing research outcomes. It can be claimed that the reduction of the sample size does not necessarily affect the outcomes of research. However, it is necessary to carefully establish the degree of this reduction since there is a “threshold” above which the findings might lose their coherence and validity.

## 5. Conclusions

The present research aimed to investigate the modifications of the outcomes of a study in the context of applied neurosciences when the sample size was reduced, specifically up to 16 subjects from an original experimental sample of 36 subjects. Three different neurophysiological indexes have been considered, two based on EEG and one on autonomic—GSR and HR—activities, during four different tasks of decreasing time duration, i.e., from 30 to 15 s. This study highlighted how, for longer tasks (30 s), sample size decrease up to 24 participants did not dramatically affect the results, i.e., the computed neurophysiological indexes kept significant correlation with the same indexes computed on the whole population, of course with the compromise of increasing mean error and standard deviation. Under this “threshold” (24 subjects), results began to lose statistical power (no significant correlations) and measure variability (MSE and STD) still increased. These effects increased in terms of magnitude as short as the task was; in fact, with 15-long-tasks, the results suggested to consider 32 as the minimum sample size.

In the light of these results, although further investigation is required to assess these findings as generalisable, it is important to underline that, even in those applied research situations where it becomes difficult to have large sample sizes, it may still be unwise to conduct research with too small a sample because the results could be not representative of the population.

## Figures and Tables

**Table 1:** Number of significant correlations (#s.c.) and eventual decreasing as percentage of the total number of combinations (630), median ± std of rho, MSE, and STD for the three indices (I1, I2, I3) and the five subgroups of subjects (32, 28, 24, 20, 16) during SPOT1.

SPOT1(30 s)												
	#s.c. (*p* < 8 × 10^−5^)			Rho			MSE			STD		
	I1	I2	I3	I1	I2	I3	I1	I2	I3	I1	I2	I3
**32**	**630** *	**630** *	**630** *	0.96 ± 0.02	0.94 ± 0.02	0.97 ± 0.01	0.012 ± 0.01	0.014 ± 0.01	0.001 ± 0.0005	0.12 ± 0.02	0.13 ± 0.01	0.03 ± 0.003
**28**	**630** *	**630** *	**630** *	0.91 ± 0.04	0.89 ± 0.05	0.94 ± 0.02	0.029 ± 0.02	0.032 ± 0.02	0.002 ± 0.001	0,18 ± 0.03	0,19 ± 0.02	0.05 ± 0.004
**24**	628 (−0.3%)	617 (−2.1%)	**630** *	0.87 ± 0.06	0.84 ± 0.07	0.90 ± 0.03	0,05 ± 0.03	0,053 ± 0.03	0,004 ± 0.002	0.23 ± 0.04	0.23 ± 0.03	0.07 ± 0.007
**20**	597 (−5.2%)	538 (−14.6%)	**630** *	0.81 ± 0,08	0.76 ± 0.10	0.86 ± 0.04	0,079 ± 0.06	0,089 ± 0.05	0,007 ± 0.003	0.30 ± 0.05	0.31 ± 0.04	0.09 ± 0.007
**16**	479 (−23.9%)	393 (−37.6%)	618 (−1.9%)	0.74 ± 0.12	0.69 ± 0.12	0.79 ± 0.06	0.128 ± 0.08	0.136 ± 0.08	0.011 ± 0.005	0.37 ± 0.06	0.38 ± 0.04	0.11 ± 0.01

**Table 2:** Number of significant correlations (#s.c.) and eventual decreasing as percentage of the total number of combinations (630), median ± std of rho, MSE, and STD for the three indices (I1, I2, I3) and the five subgroups of subjects (32, 28, 24, 20, 16) during SPOT2.

SPOT2 (30 s)												
	#s.c. (*p* < 8 × 10^−5^)			Rho			MSE			STD		
	I1	I2	I3	I1	I2	I3	I1	I2	I3	I1	I2	I3
**32**	**630** *	**630** *	**630** *	0.98 ± 0.007	0.95 ± 0.02	0.94 ± 0.02	0.02 ± 0.01	0.02 ± 0.01	0.001 ± 0.00	0.14 ± 0.02	0.13 ± 0.02	0.03 ± 0.004
**28**	**630** *	**630** *	**630** *	0.96 ± 0.01	0.9 ± 0.04	0.88 ± 0.04	0.04 ± 0.01	0.04 ± 0.02	0.002 ± 0.00	0.21 ± 0.02	0.19 ± 0.03	0.05 ± 0.006
**24**	**630** *	620 (−1.6%)	598 (−5.1%)	0.94 ± 0.03	0.84 ± 0.07	0.81 ± 0.07	0.07 ± 0.03	0.07 ± 0.03	0.004 ± 0.001	0.27 ± 0.04	0.26 ± 0.05	0.07 ± 0.008
**20**	622 (−1.2%)	540 (−14.3%)	492 (−21.9%)	0.91 ± 0.06	0.78 ± 0.10	0.74 ± 0.11	0.12 ± 0.05	0.11 ± 0.05	0.007 ± 0.003	0.34 ± 0.04	0.32 ± 0.06	0.08 ± 0.01
**16**	581 (−7.8%)	415 (−34.1%)	340 (−46%)	0.86 ± 0.08	0.70 ± 0.13	0.67 ± 0.14	0.18 ± 0.07	0.17 ± 0.07	0.01 ± 0.004	0.42 ± 0.06	0.40 ± 0.07	0.1 ± 0.02

**Table 3:** Number of significant correlations (#s.c.) and eventual decrease as percentage of the total number of combinations (630), median ± std of rho values for the three indices (I1, I2, I3), and the five subgroups of subjects (32, 28, 24, 20, 16) during SPOT3 and SPOT4.

SPOT3 (20 s)							SPOT4 (15 s)					
	#s.c. (*p* < 8 × 10^−5^)			Rho			#s.c. (*p* < 8 × 10^−5^)			Rho		
	I1	I2	I3	I1	I2	I3	I1	I2	I3	I1	I2	I3
**32**	**630** *	**630** *	**630** *	0.98 ± 0.01	0.95 ± 0.03	0.98 ± 0.01	**630** *	623 (−1.1%)	**630** *	0.96 ± 0.026	0.96 ± 0.04	0.97 ± 0.01
**28**	**630** *	607 (−3.6%)	**630** *	0.95 ± 0.03	0.88 ± 0.06	0.95 ± 0.02	567 (−10%)	548 (−13%)	611 (−3%)	0.92 ± 0.04	0.93 ± 0.05	0.94 ± 0.02
**24**	618 (−1.9%)	515 (−18.2%)	628 (−0.3%)	0.92 ± 0.05	0.83 ± 0.08	0.92 ± 0.04	416 (−33.9%)	427 (−32.2%)	508 (−19.3%)	0.87 ± 0.09	0.87 ± 0.13	0.89 ± 0.04
**20**	577 (−8.4%)	340 (−46%)	567 (−10%)	0.88 ± 0.07	0.76 ± 0.13	0.87 ± 0.06	295 (−53.2%)	288 (−54.3%)	334 (−46.9%)	0.80 ± 0.12	0.81 ± 0.19	0.84 ± 0.06
**16**	494 (−21.6%)	222 (−64.7%)	414 (−34.3%)	0.83 ± 0.10	0.70 ± 0.16	0.81 ± 0.07	166 (−73.7%)	180 (−71.4%)	207 (−67.1%)	0.74 ± 0.17	0.77 ± 0.24	0.78 ± 0.08

**Table 4:** Number of significant correlations (#s.c.) and eventual decreasing as percentage of the total number of combinations (630) for the three indices (INDEX1, INDEX2, INDEX3) and the five subgroups of subjects (32, 28, 24, 20, 16) during SPOT1 (30 s), SPOT2 (30 s), SPOT3 (20 s), and SPOT4 (15 s).

	INDEX1				INDEX2				INDEX3			
	#s.c. (*p* < 8 × 10^−5^)				#s.c. (*p* < 8 × 10^−5^)				#s.c. (*p* < 8 × 10^−5^)			
	30 s	30 s	20 s	15 s	30 s	30 s	20 s	15 s	30 s	30 s	20 s	15 s
**32**	**630** *	**630** *	**630** *	**630** *	**630** *	**630** *	**630** *	623 (−1.1%)	**630** *	**630** *	**630** *	**630** *
**28**	**630** *	**630** *	**630** *	567 (−10%)	**630** *	**630** *	607 (−3,6%)	548 (−13%)	**630** *	**630** *	**630** *	611 (−3%)
**24**	628 (−0.3%)	**630** *	618 (−1.9%)	416 (−33.9%)	617 (−2.1%)	620 (−1.6%)	515 (−18.2%)	427 (−32.2%)	**630** *	598 (−5.1%)	628 (−0.3%)	508 (−19.3%)
**20**	597 (−5.2%)	622 (−1.2%)	577 (−8.4%)	295 (−53.2%)	538 (−14.6%)	540 (−14.3%)	340 (−46%)	288 (−54.3%)	**630** *	492 (−21.9%)	567 (−10%)	334 (−46.9%)
**16**	479 (−23.9%)	581 (−7.8%)	494 (−21.6%)	166 (−73.7%)	393 (−37.6%)	415 (−34.1%)	222 (−64.7%)	180 (−71.4%)	618 (−1.9%)	340 (−46%)	414 (−34.3%)	207 (−67.1%)

## Data Availability

The aggregated data analysed in this study might be available on request from the corresponding author.
